# CRH promotes *S. pneumoniae* growth *in vitro* and increases lung carriage in mice

**DOI:** 10.3389/fmicb.2015.00279

**Published:** 2015-04-08

**Authors:** Colette G. Ngo Ndjom, Harlan P. Jones

**Affiliations:** ^1^Department of Molecular and Medical Genetics, University of North Texas Health Science Center, Fort Worth, TXUSA; ^2^Center for Biotechnology Education, Krieger School of Arts and Sciences, Johns Hopkins University, Baltimore, MDUSA

**Keywords:** Corticotropin releasing hormone, *Streptococcus pneumoniae*, pathogenicity, hormones, virulence, commensal, respiratory

## Abstract

*Streptococcus pneumoniae (S. pneumoniae*), a commensal across the nasal passages, is responsible for the majority of infectious pneumonia cases worldwide. Previous studies have shown that hormonal factors may be influential in regulating *S. pneumoniae’s* transition from a non-pathogen to a pathogenic state. The current study investigated the effects of corticotropin-releasing hormone (CRH), a peptide hormone involved in stress, on the pathogenicity of *S. pneumoniae*. Mice were infected with CRH-treated *S. pneumoniae* via intranasal route, showing an increase in pulmonary bacterial burden. We also quantified *S. pneumoniae*’s response to CRH through limited serial dilutions and growth curve analysis. We demonstrated that CRH promotes *S. pneumoniae* titer-dependent proliferation, as well as accelerates log-phase growth. Results also showed an increase in pneumococcal-associated virulence protein A virulence gene expression in response to CRH. These results demonstrate a role for CRH in *S. pneumoniae* pathogenicity, thus implicating CRH in mediating the transition of *S. pneumoniae* into a pathogenic state.

## Introduction

*Streptococcus pneumoniae* (*S. pneumoniae*) contributes to the highest morbidity and mortality rates worldwide (Laupland and Church, 2014; Oishi et al., 2014). Approximately $4.9 billion dollars in healthcare cost is attributed to pneumonia in the United States (US; Reynolds et al., 2014). The magnitude of this problem is underscored by the significant mortality risks among hospitalized patients ranging between 10 and 25% and increasing to 50% among patients 65 years and older (Reynolds et al., 2014). Although increased antibiotic resistance plays a role, it does not entirely explain the problem. For example, penicillin-resistance accounts for the majority of pneumococcal resistance but attributes to only 4% of the total estimated healthcare costs (Laxminarayan, 2014). Based on these statistics it is clear that a gap in knowledge exists, limiting our understanding of the epidemiology of pneumonia caused by *S. pneumoniae* infection.

Among the 92 known serotypes of *S. pneumoniae*, few are considered virulent. Moreover, invasive serotypes are not contagious under conditions within their disease states (e.g., pneumonia, otitis media, and sepsis; Caierão et al., 2014; Del Amo et al., 2014; Feldman and Anderson, 2014). Rather, transmission of disease stems from the reservoir of resident asymptomatic pneumococci along nasal passages, which, through unknown mechanisms, acquires pathogenic status. It is believed that as a commensal, *S. pneumoniae*’s pathogenicity is largely influenced by complex symbiotic relationships within the host including commensal competition, nutritional status, metabolic stress, mucosal-associated immune responses, and hormonal signals (Alcaide et al., 2012; Bailey, 2014; Cameron et al., 2014; Frydenborg et al., 2014; Ishak et al., 2014). Neuropeptides in particular, have been shown to mediate commensal ecosystems that impact disease susceptibility (Freestone et al., 2008; Schoen et al., 2014). Most of what is understood linking microbial species’ response to stress hormones relates to the neuroendocrine gut mucosal system and its interaction with enteric bacterial species (Freestone and Lyte, 2008; Lyte, 2014). However, mechanisms of disease between hormonal factors and respiratory bacterial commensals remain elusive.

Current knowledge of microorganisms’ response to neuroendocrine factors largely stem from studies defining how dopaminergic, catecholaminergic inotropes and cholinergic pathways impact gut-associated microbes (Freestone and Lyte, 2008). For example, catecholamines have been shown to stimulate toxin release, motility, and attachment by *Escherichia coli* O157:H7, *Vibrio parahaemolyticus* and other pathogens (Freestone et al., 2007a). However, far less is known regarding hormonal signals’ impact on infectious disease across the respiratory tract. Two recent studies have examined the role of catecholamine responses by *S. pneumoniae*. Specifically, Sandrini et al. (2014) demonstrated that norepinephrine (NE) could promote pneumococcal growth and biofilm formation. In addition, Gonzales et al. (2013) showed that NE reduced lung adherence through iron binding as a potential mechanism. This is consistent with the hypothesis that bacterial species along the respiratory tract are responsive to hormonal factors as a potential inducer of pathogenicity. Due to high mortality risks associated with respiratory disease caused by *S. pneumoniae*, one might expect as a critical need to understand the role of neuroendocrine responses that mediate its pathogenicity and that of other respiratory commensal organisms.

Corticotropin-releasing hormone (CRH) is a 41-amino acid peptide produced in the hypothalamus and throughout the brain where it plays a role in behavior and autonomic responses to stress (Clynen et al., 2014; Gold, 2014; Kolasa et al., 2014). CRH is also defined by its induction of peripheral glucocorticoid (e.g., cortisol in humans and corticosterone in mice) secretion, known to suppress immune function (Quintanar and Guzman-Soto, 2013; Uchoa et al., 2014). In contrast, CRH production also occurs at peripheral sites and appears to be involved in inflammatory conditions associated with diseases such as rheumatoid arthritis, cancer, colitis, and asthma (Vasiadi et al., 2012; Clynen et al., 2014). Using a murine model of aversive stress and *S. pneumoniae* infection, we have previously demonstrated the expression of CRH in the lung, as well as its impact on host pulmonary cellular immune and inflammatory responses (Gonzales et al., 2008; Kim et al., 2011). The purpose of the current study was to test whether CRH directly impacts *S. pneumoniae* virulence. Findings presented here demonstrate that CRH directly increases bacterial growth, a key characteristic of invasiveness. In support of the observed increase in bacterial growth, we also demonstrated influences of CRH on the regulation of pavA, a virulence protein expressed by *S. pneumoniae* associated with the mediation of immune and inflammatory responses. Moreover, infection of mice with CRH-treated *S. pneumoniae* resulted in greater bacterial carriage in the lung.

## Materials and Methods

### Bacterial Strains

*Streptococcus pneumoniae* strain #6301 (ATCC, Manassas, VA, USA) was used in all experiments. Prior to use, *S. pneumoniae* was maintained in 30% glycerol frozen stock solutions (–80^∘^C).

### Corticotropin-Releasing Hormone (CRH)

Human/rat recombinant CRH (Sigma–Aldrich, St. Louis, MO, USA) was used in all experiments. CRH stock solutions were stored at –20^∘^C in 20 μl aliquots until use.

### Urocortin (UCN)

Urocortin (Sigma–Aldrich, St. Louis, MO, USA), a related CRH homolog peptide, was used as a relevant negative control. UCN stock solutions were stored at –20^∘^C in 2 ml aliquots until use.

### Mice

Female CD1 strain (Harlan Laboratories, Houston, TX, USA) between six and 8 weeks of age were used in all *in vivo* experiments. Mice were maintained in sterile conditions and provided food and water *ad libitum*. For infection studies, mice were anesthetized by intraperitoneal injection of ketamine/xylazine solution (50 mg/kg ketamine and 16 μg/kg xylazine for a total volume of 0.2 ml). The University of North Texas Health Science Center’s Institutional Animal Use Committee (IACUC) approved all studies.

### *In Vivo* Determination of CRH-Treated *S. pneumoniae* Pathogenesis

Briefly, frozen stock cultures were spread onto blood agar plates and incubated for 18 h at 37^∘^C and 5% CO_2_ to achieve mid-log phase growth. Cultures were suspended in a 50/50 Brain–Heart Infusion (BHI; EMD Chemicals Inc., Darmstadt, Germany) and Phosphate Buffered Saline (PBS; Life Technologies, Carlsbad, CA, USA) broth mixture. Subsequent bacterial suspensions were adjusted to an optical density (OD) of 1.0 containing approximately 1 × 10^8^ bacterial cells.

Prior to infection, bacterial cells were diluted to 1 × 10^5^ cells of *S. pneumoniae* and were exposed to 2.1 × 10^-4^ mM/μl of CRH overnight at 37^∘^C and 5% CO_2._ *S. pneumoniae* was collected and diluted to an infection dose of 2.0 × 10^5^ CFUs (LD_50_). Subsequently, anesthetized mice (*N* = 5/group) were administered CRH-treated (CRH-Sp) or untreated *S. pneumoniae* (Sp; LD_50_) by intranasal route. Eighteen hours following infection, anesthetized mice were euthanized to compare bacterial carriage in lungs of CRH-Sp versus Sp-infected mice as previously described. Specifically, lungs were harvested and homogenized in sterile cold PBS (Life Technologies, Carlsbad, CA, USA). Ten-fold serial dilutions of lung homogenates were plated in duplicates onto blood agar plates and incubated at 37^∘^C overnight. Colonies on plates were enumerated, and the results were expressed as log_10_ CFU per μl.

### Quantitation of *S. pneumoniae* in Response to CRH by Limited Dilution CFU Analysis

*Streptococcus pneumoniae* strain #6301 (ATCC, Manassas, VA, USA) was grown overnight to achieve mid-log phase cultures on Blood Agar plates. *S. pneumoniae* was collected and suspended in a 50/50 BHI and PBS broth mixture. Ten-fold dilutions of a starting cell number of 1.3 × 10^6^ were seeded in sterile 96-well flat bottom plates in the presence or absence of CRH at concentrations of 2.1 × 10^-4^mM/μl and 4.0 × 10^-4^ mM/μl, respectively. In addition, 4.0 × 10^-4^ mM/μl UCN was introduced to bacterial cultures to serve as a negative control. Aliquots (8 μl) of bacterial suspensions with or without CRH or UCN were plated on blood agar plates and incubated at 37^∘^C overnight. Colonies on plates were enumerated, and the results were expressed as log_10_ CFU per μl. All experiments were performed in duplicate.

### Growth Curve Determination in Response to CRH

Bacterial stock of *S. pneumoniae* was prepared by adding 50 μl of bacteria from frozen stock to 1950 μl of sterile BHI broth. The mixture was incubated overnight at 37^∘^C (5% CO_2_). Twenty-four hours later absorbance of the overnight culture was determined and adjusted to 0.2 at OD_600_ in BHI. A series of duplicate tubes were prepared containing 1950 μl and 50 μl of *S. pneumoniae* in the presence or absence of 2.1 × 10^-4^mM/μl CRH and incubated at 37^∘^C (5% CO_2_). Duplicate absorbance readings (OD_600_) of bacterial cultures were taken every 30 min. over a 9 h time period. Absorbance readings were plotted against time to generate bacterial growth curves.

### Gene Expression Profiling by Quantitative Real-Time Polymerase Chain Reaction (qRT-PCR)

*Streptococcus pneumoniae* from freezer stock was plated overnight at 37^∘^C (5% CO_2_). After an 18 h of incubation, bacteria were collected and suspended in BHI and the absorbance was read and adjusted to 1 (OD_600_ = 1.0). Bacterial cells were incubated overnight at 37^∘^C (5% CO_2_) in presence or absence of 2.1 × 10^-4^ mM/μl CRH.

#### Bacterial RNA Extraction

Eighteen hours after incubation, *S. pneumoniae* from representative experimental conditions were collected in separate labeled tubes using sterile BHI broth. Absorbance of each sample was read and adjusted to an OD_600_ = 1.0, representing 10^8^–10^9^ cell density. RNA extraction from bacterial cells was performed using a RiboPure RNA Purification Kit (Life Technologies, Carlsbad, CA, USA).

#### Reverse Transcription and qRT-PCR

cDNA was generated using a starting Total RNA concentration of 1 μg per reaction and MLV (Molony murine leukemia virus) reverse transcriptase (Promega Corp., Madison, WI, USA). After cDNA synthesis, real-time PCR was performed using SYBR green-based amplification techniques. PCR was performed in a 20 μl reaction volume using the StepOne system (Applied Biosystems Inc., Foster City, CA, USA). The expression of the housekeeping gene 16s rRNA was used as an internal control to normalize target gene expression between samples. Pneumococcal adherence and virulence factor A (pavA) gene expression in bacterial cells was calculated using the following formula:

$\Delta \Delta CT=\Delta CT^{\left(t\arg et\;\;gene\right)}-\Delta CT^{\left(16s\;\;rRNA\right)}$ (Kim et al., 2011)

Data was calculated by subtracting δδ^CT^ of control group from the CRH-treated group for each target gene. Data was expressed as the fold difference between pavA by normalizing the expression of untreated target genes as 1. Selected target and house keeping gene primer sets; pavA and 16s rRNA were purchased from Life Technologies, Carlsbad, CA, USA (**Table 1**).

**Table 1:** List of primer sequences and target gene.

	Sequence
**House keeping Gene^1^**
16S rRNA-Forward (5′–3′)	TGAGTTAACCGTAAGGAGCCA
16S rRNA-Reverse (3′–5′)	TCACCCCAATCATCTATCCCA
**pavA (Pneumococcal adherence and virulence factor A)^2^**
Forward (5′–3′)	GGTCGCATCCAGAAAATC
Reverse (3′–5′)	AGAAAGGAGCAGGCGATG

*^1^Gonzales et al. (2013); ^2^Cope et al. (2011).*

### Statistical Analysis

Statistical analysis was performed using GraphPad Prism Version 6.0 (GraphPad Software, San Diego, CA, USA). For multi-experimental group analysis, data were subjected to one-way and two-way ANOVA (analysis of variance) followed by *post hoc* tests (Newman–Keuls and Bonfferoni) for group differences. Other results were analyzed using *t*-test where indicated. All data are expressed as means ± SEM. A *P*-value of ≤ 0.05 was considered significant.

## Results

### CRH-Treated *S. pneumoniae* Increases Lung Carriage

**FIGURE 1:**
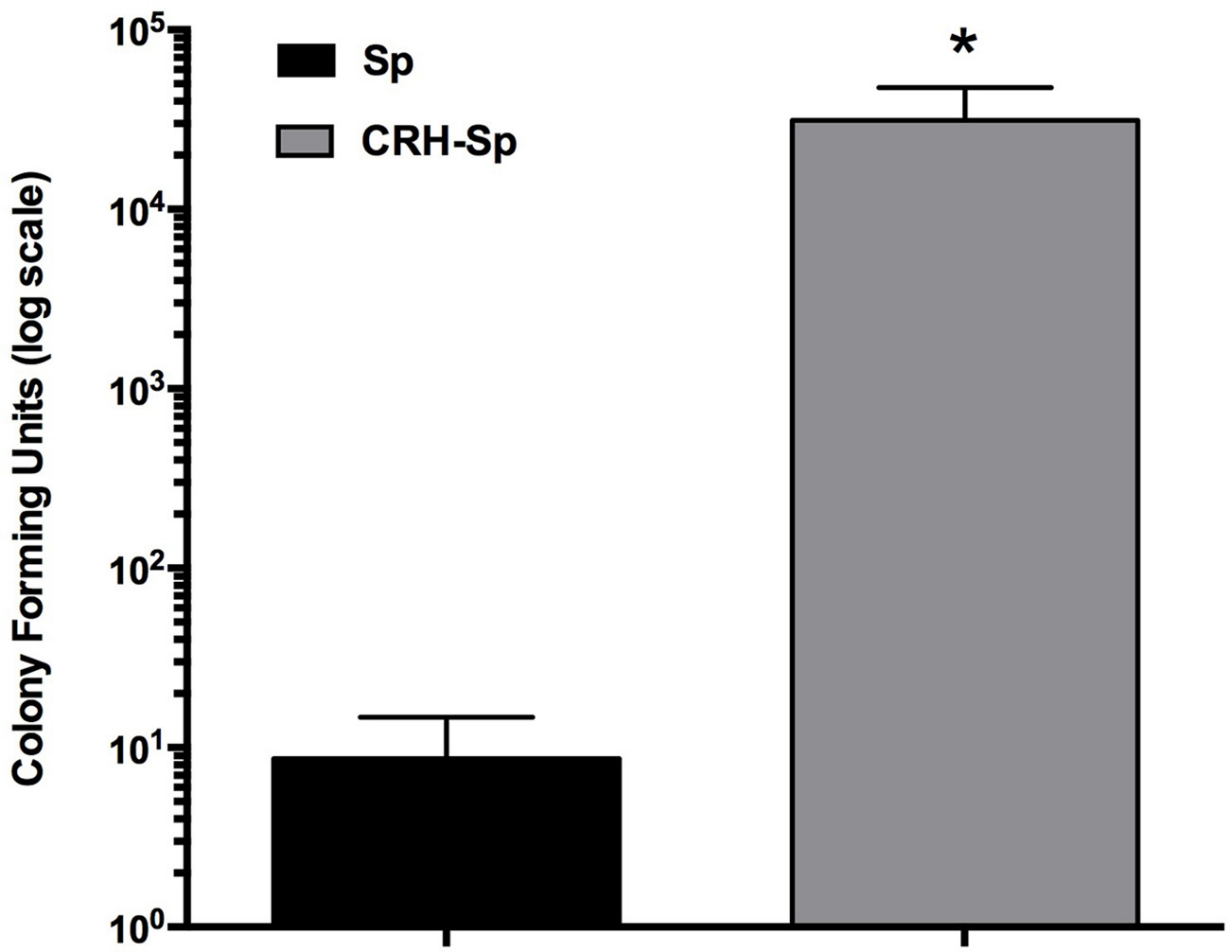
**Corticotropin-releasing hormone (CRH)-treated *Streptococcus pneumoniae* increases mice lung carriage.** We assessed whether or not exposing mice to *S. pneumoniae* (10^5^ cells), previously treated or untreated with CRH, would result in bacterial burden for the mice. Mice (*N* = 5) treated with previously exposed to CRH bacteria (2.0 × 10^5^ cells) showed an increase in lung carriage compared to the unexposed group. Bar graphs represent the mean ± SE of (*N* = 5) the number of CFUs in lungs. Asterisks (^∗^) indicate significant (*P* ≤ 0.05) differences between experimental groups.

We determined whether exposing *S. pneumoniae* to CRH would impact its propensity to induce pulmonary infection. *S. pneumoniae* cultures were exposed to CRH (2.1 × 10^-4^mM/μl) and used for nasal infection. Bacterial colonization in the lungs of mice was significantly higher (*P* ≤ 0.05) in mice exposed CRH-treated *S. pneumoniae* (CRH-Sp) compared to untreated *S. pneumoniae* (Sp; **Figure 1**).

### CRH Promotes *S. pneumoniae* Titer-Dependent Proliferation

**FIGURE 2:**
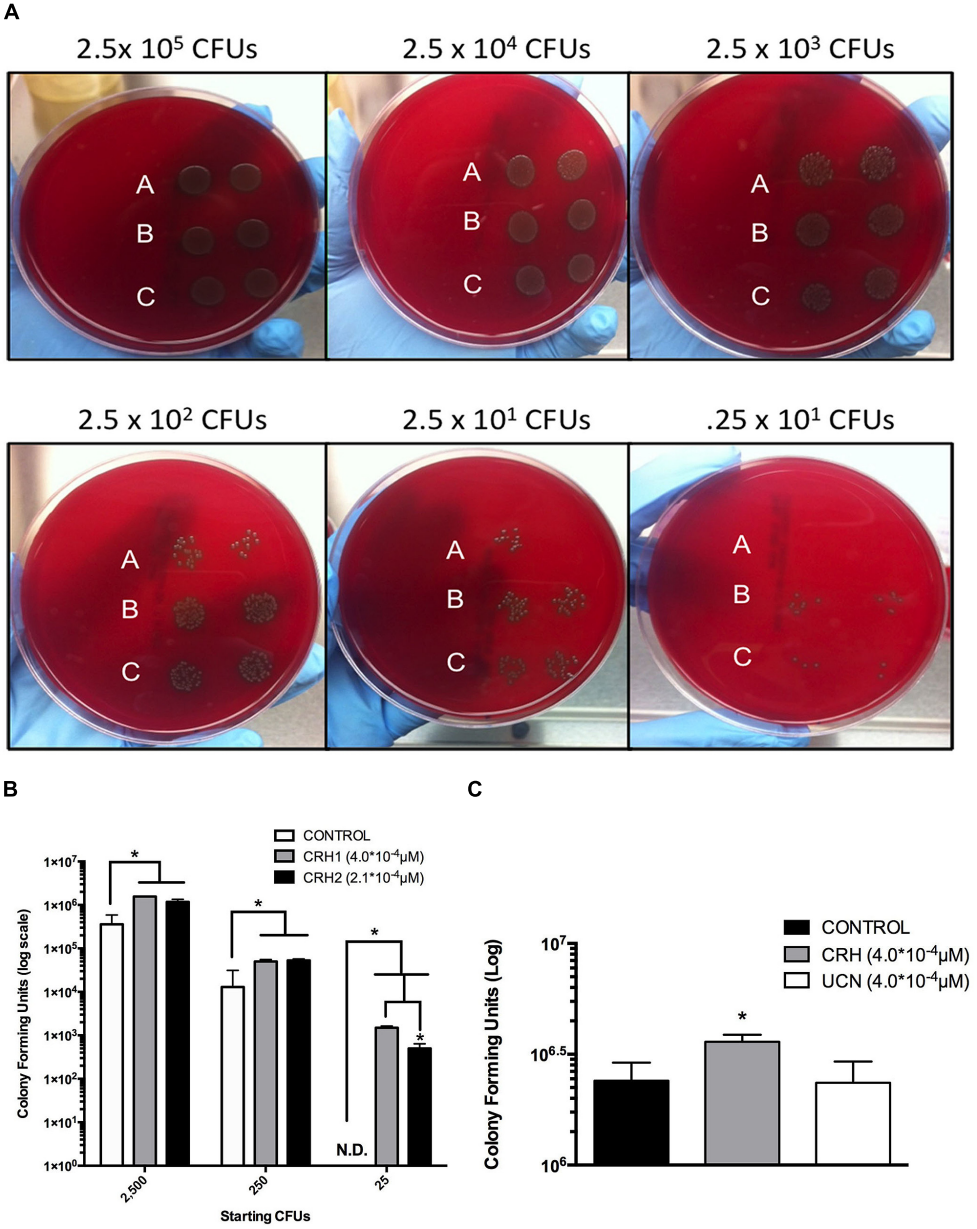
**Titer-dependent effects of CRH on *S. pneumoniae* colony formation.** Ten-fold serial dilutions of *S. pneumoniae* were incubated in the absence **(A)** or presence of two concentrations of CRH, 2.1 × 10^-4^ μM **(B)** and 4.0 × 10^-4^ μM, **(C)** and plated on blood agar plates. Data shown is representative of two independent experiments **(A)**. **(B)** Represents quantitation of the aforementioned results in **(A)**. Bars represent mean (*N* = 3) ± SE. Asterisks (^∗^) indicate significant (*P* ≤ 0.05) differences between experimental groups. White bars denote unexposed *S. pneumoniae*, gray and black bars denote *S. pneumoniae* exposed to two different CRH concentrations, 4.0 × 10^-4^ μM and 2.0 × 10^-4^ μM, respectively **(B)**. **(C)** Highlights the specificity of CRH compared to its homolog UCN. Bars represent mean (*N* = 3) ± SE. Asterisks (^∗^) indicate significant (*P* ≤ 0.05) differences between experimental groups.

The above results suggested that CRH directly promotes the pathogenicity of *S. pneumoniae* by increased colonization in the lung. We assessed the effect of CRH on *S. pneumoniae* proliferation controlling for bacterial titer and variation in CRH concentration. *S. pneumoniae* colony forming units (CFUs) were significantly higher (*P* ≤ 0.05) given exposure to both concentrations of CRH at each titer of *S. pneumoniae*. In contrast to un-treated cultures of *S. pneumoniae*, CRH significantly sustained detection of CFUs at the lowest titer (8th-fold dilution; **Figures 2A,B**). To confirm specificity of CRH’s effect, additional experiments were performed whereby *S. pneumoniae* was exposed to the CRH homolog UCN. Lower concentrations of UCN (2.1 × 10^-4^mM/μl) were also used in testing. No differences in CFUs were observed in the presence of UCN at different concentrations compare to untreated *S. pneumoniae* (**Figure 2C**).

### CRH Accelerates Log-Phase Growth of *S. pneumoniae* Growth

**FIGURE 3:**
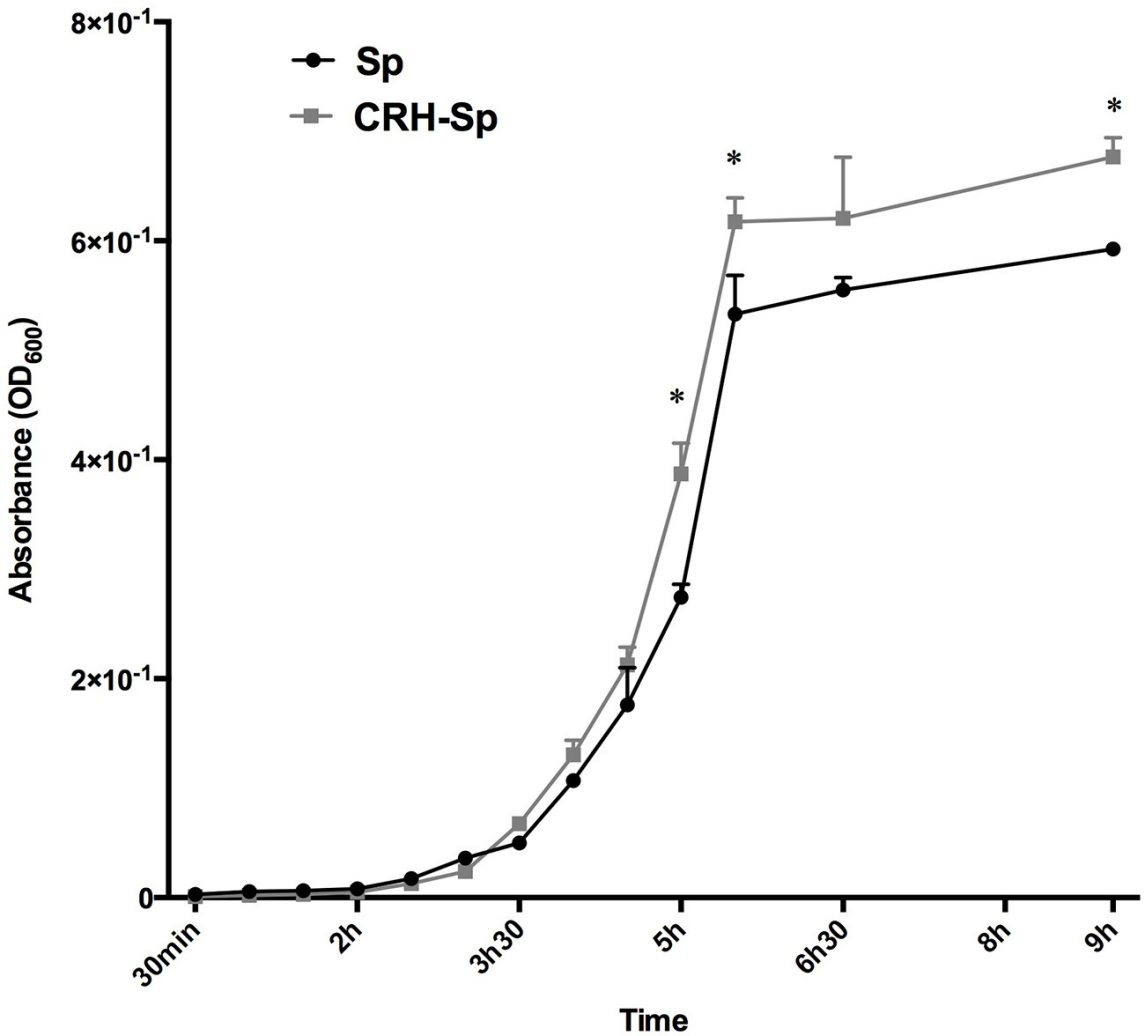
**Corticotropin-releasing hormone accelerates log-phase growth of *S. pneumoniae*.** *S. pneumoniae*, CRH exposed (2.1 × 10^-4^ μM/μl) vs. non-exposed, growth analysis was performed over a period of 9 h. Asterisks (^∗^) indicate significant (*P* ≤ 0.05) differences observed in the final bacterial mass during stationary phase. All experiments were performed in duplicates.

We compared the bacterial growth curve in presence and absence of CRH. A significant difference (*P* ≤ 0.05) was observed in bacteria exposed to CRH during log phase growth. In addition, significant differences in the final bacterial mass were observed during stationary growth phase (**Figure 3**).

### CRH Increases Pneumococcal Adherence and Virulence Factor A (pavA) mRNA Gene Expression by *S. pneumoniae*

**FIGURE 4:**
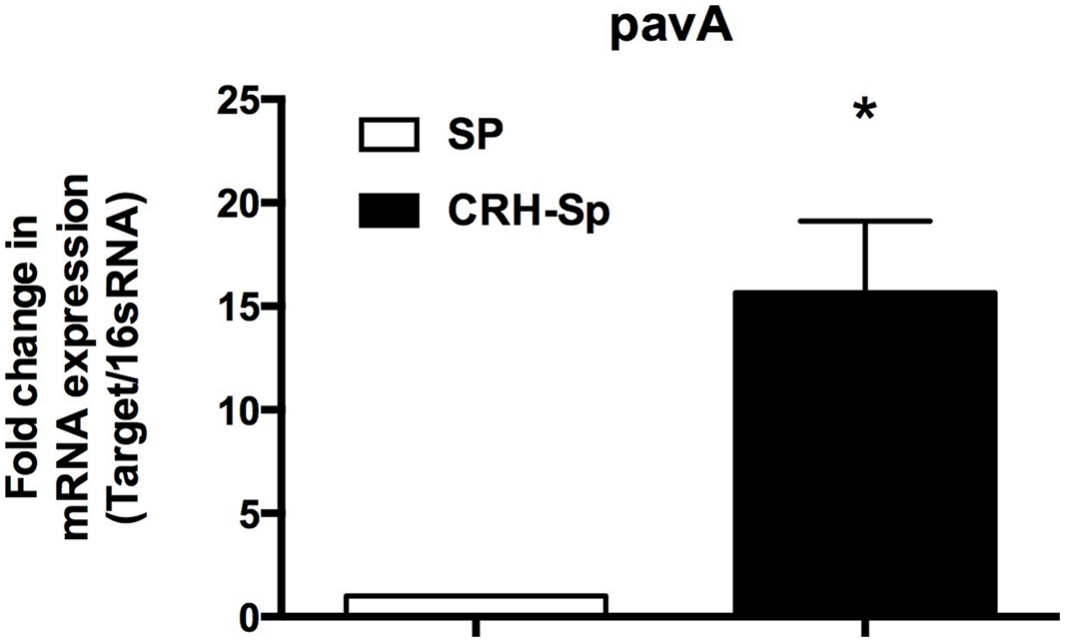
***Streptococcus pneumoniae* adhesion and virulence factors increase in the presence of CRH.** pavA gene expression was determined by quantitative realtime PCR analysis of *S. pneumoniae’s* mRNA exposed to CRH (*N* = 3). The analysis showed overexpression of the bacterium’s adhesion and virulence gene (pavA) when compared to control genes. Asterisks (^∗^) indicate significant (*P* ≤ 0.05) difference between CRH treated (CRH-Sp) and non-CRH treated (Sp) bacterial groups.

PavA gene expression is found to modulate bacterial adhesion, invasion, and inflammation associated with septicemia (Pracht et al., 2005). **Figure 4** demonstrates a significant (*P* ≤ 0.05) increase in pavA mRNA expression by *S. pneumoniae* exposed to CRH, compared to untreated controls as determined by quantitative realtime PCR.

## Discussion

This study expands upon an emerging area of research that seeks to define hormonal signals as determinants for pathogenic transformation of commensal bacterial species (Freestone, 2013; Lyte, 2014). We demonstrate how CRH, a hormonal mediator of central and peripheral neuro-immune axes, works in mediating virulence-associated factors of the upper respiratory tract commensal *S. pneumoniae*. Moreover, our previous studies established the role of CRH in regulating experimental pneumonia and sepsis caused by *S. pneumoniae* (Gonzales et al., 2008; Kim et al., 2011), implicating it in mediation of host cellular immune and inflammatory responses. Research defining CRH and other hormonal factors as direct modifiers of respiratory commensal species remain unknown.

A commensal organism’s ability to sense a change in its environment is essential for its survival. Prevalent hormonal stress signals identified to influence bacterial phenotype and function include catecholamines, NE and epinephrine (Freestone and Lyte, 2010). Studies demonstrated that *in vitro*, NE and epinephrine promote microorganism’s growth, metabolic potential, nutritional status, and biofilm formation (Neal et al., 2001; Lyte et al., 2003; Freestone et al., 2007b; Yang et al., 2014b) furthermore suggesting that catecholamines, and possibly other hormonal stress factors, are influential regulators of microbial virulence (Li et al., 2009; Lucas et al., 2012; Pande et al., 2014). However, aforementioned hormonal effects have been primarily a focus of bacterial species resident in gut mucosa.

Previous studies have been associated with the activation of stress factors with disease pathogenesis along respiratory tissues (Lim et al., 2014; Runeson-Broberg and Norback, 2014; Tomljenovic et al., 2014; Yang et al., 2014a). Utilizing an experimental model of aversive stress and *S. pneumoniae* infection, we demonstrated CRH’s ability to regulate disease severity by controlling cellular immune and inflammatory responses (Kim et al., 2011). Notably, our studies demonstrated that inhibiting CRH from binding its target receptor CRHR1 caused increased pneumonia and sepsis. We thus hypothesized that increasing peripheral CRH levels allows for increased exposure to *S. pneumoniae*, potentially impacting its pathogenicity. In the current study*,* we tested the possible direct effects imparted by CRH on the propensity of *S. pneumoniae* to cause disease, to influence bacterial growth and to modulate pavA gene expression. As shown in **Figure 1**, exposing *S. pneumoniae* cultures to CRH prior to experimental infection produced greater pulmonary bacterial burden compared to infection with untreated cultures. This finding suggested that independent of CRH’s impact on host cellular immune function, disease severity could be elicited from *S. pneumoniae* being directly exposed to CRH; thus supporting the potential for *S. pneumoniae* to recognize and respond to CRH for purposes of modulating its phenotype.

Possible mechanisms through which CRH could be imparting its function were determined by testing its effects on key indicators of bacterial virulence (e.g., microorganisms’ growth). We determined that CRH enhanced the growth phase kinetics of *S. pneumoniae* (**Figure 3**), reinforcing earlier studies demonstrating hormonal factors’ ability to modulate bacterial growth (Freestone et al., 2007b; Seeley et al., 2013; Pande et al., 2014). The ability of CRH to promote colony formation further substantiated its role in growth-associated virulence at significantly lower bacterial titers (**Figures 2A-C**). Ongoing studies using a clinical invasive serotype 3 pneumococcal strain were more invasive in the presence of CRH interpreted by its ability to reestablish secondary colony formation from primary biofilms (Thapa et al., unpublished data). These findings suggest a role for CRH in regulating mechanisms of invasiveness associated with high risk for pneumonia and sepsis.

The studies presented here highlight for the first time the ability of CRH to serve as a catalyst for *S. pneumoniae* virulence. Mechanisms of action have been identified as basis for *S. pneumoniae’s* response to stress hormonal signals. Catecholamines play a role in the process of Fe-transport across bacterial membranes and making Fe valence available for enriched metabolic processes (Freestone et al., 2000, 2003). NE, a known siderophore, enhances iron availability to *S. pneumoniae*, thus promoting bacterial growth and virulence (Kinney et al., 2000; Gonzales et al., 2013). Whereas siderophore processing cascades of Gram-negative species have been understood (Seeley et al., 2013), they remain unknown for the Gram-positive bacterial species. Future studies should address how CRH is sensed and processed by *S. pneumoniae* and other bacterial species.

The inability to tailor inflammatory responses hinders significant advances to improve disease outcomes associated with infectious pneumonia. Despite the current use of systemic corticosteroids as standard adjunctive treatment to reduce inflammatory reactions in cases of pneumonia (Steel et al., 2013; Sibila et al., 2014), mortality and morbidity rates remain high (Lin et al., 2010; Moon et al., 2012; Peterkovic et al., 2012), raising questions of their benefit (Ramsey and Gorman, 2014; Sibila et al., 2014). Stress-induced release of endogenous glucocorticoids or other hormones may negatively impact adjunctive corticosteroid use. Currently there are few studies demonstrating the potential relationships between glucocorticoids and bacterial pathogenicity. *Pseudomonas aeruginosa* was found to produce a protease factor that inhibits cortisol-binding globulin as a mechanism of blocking plasma cortisol transport (Simard et al., 2014). In addition, Verbrugghe et al. (2011) showed that cortisol can directly impact bacterial functioning. They demonstrated that *Salmonella typhimurium’s* proliferation was increased within macrophages exposed to cortisol in pigs, but not in the presence of catecholamines (Verbrugghe et al., 2011). In the current study, we found that CRH increased pavA (modulator of bacterial adherence and inflammation) gene expression by *S. pneumoniae* (**Figure 4**), thus highlighting the role of CRH in bacterial-associated inflammatory responses. While mechanisms defining interactions between the central nervous and immune systems have been proved important for host susceptibility and resolution of disease (Dhabhar, 2014), few therapies take into account the direct influence of stress factors on bacterial physiology. In total, our results provide evidence in support of CRH to directly influence *S. pneumoniae* virulence. However, limitations requiring further investigation such as the direct abrogation of CRH-mediated responses will be key to understanding the mechanistic relationships between CRH and its pathogenicity. Such studies could lead to novel approaches to reduce the onset of disease.

## Conflict of Interest Statement

The authors declare that the research was conducted in the absence of any commercial or financial relationships that could be construed as a potential conflict of interest.
